# The association between body composition and arterial stiffness: investigating the influence of inflammatory markers

**DOI:** 10.3389/fcvm.2026.1898248

**Published:** 2026-09-21

**Authors:** Julia Neubauer, Dennis Freuer, Marlene Haupt, Jakob Linseisen, Christine Meisinger, Simone Fischer

**Affiliations:** 1 Faculty of Social Work, Health & Nursing, University of Applied Sciences Ravensburg-Weingarten, Weingarten, Germany; 2 Epidemiology, Medical Faculty, University of Augsburg, Augsburg, Germany; 3 Faculty of Applied Social Sciences, Munich University of Applied Sciences, München, Germany

**Keywords:** arterial stiffness, augmentation index, bioimpedance analysis, inflammatory markers, mediation analysis, obesity, pulse wave velocity

## Abstract

**Background:**

Body fat distribution may influence arterial stiffness through inflammatory pathways, but evidence from relatively healthy adults and across complementary measures of arterial stiffness remains limited. This study investigated associations between bioelectrical impedance-derived body composition measures and arterial stiffness and assessed whether selected markers of inflammation and endothelial activation explained these associations.

**Methods:**

This study utilized cross-sectional baseline data from the MEGA study, involving 219 healthy non-obese (BMI < 30 kg/m^2^) and obese (BMI ≥ 30 kg/m^2^) participants with a mean age of 46 ± 12 years. Exposures were visceral fat (L), total body fat (%), fat mass index (kg/m^2^), and fat-free mass index (kg/m^2^). Outcomes were carotid-femoral pulse wave velocity (cfPWV, m/s), augmentation index (AIx, %), and central pulse pressure (cPP, mmHg). Tumor necrosis factor-alpha, interleukin-6, and vascular cell adhesion molecule-1 were evaluated as potential intermediate factors. Multivariable linear regression used robust standard errors and false-discovery-rate-adjusted p-values. Mediation analyses examined associations between body composition, arterial stiffness, and inflammatory markers, using 5,000 bootstrap samples.

**Results:**

Visceral fat, total body fat, and fat mass index were positively associated with AIx: The adjusted regression coefficients were *β* = 0.642 (95% confidence interval, 0.172–1.112; *p* = 0.032) for visceral fat, *β* = 0.186 (95% confidence interval, 0.083–0.288; *p* < 0.001) for total body fat, and *β* = 0.333 (95% confidence interval, 0.157–0.508; *p* < 0.001) for fat mass index, respectively. No bioimpedance-derived measure was independently associated with cfPWV or cPP after correction for multiple testing and no indirect associations through inflammatory markers were found.

**Conclusion:**

Higher visceral fat, total body fat, and fat mass index were associated with higher AIx, but the selected inflammatory markers did not explain these cross-sectional associations. Longitudinal studies with broader biomarker panels are needed before causal or clinical conclusions can be drawn.

## 1 Introduction

The prevalence of overweight and obesity is steadily increasing, with the World Obesity Federation estimating that over four billion people may be affected by 2035 (1). Obesity, defined by the WHO as body mass index (BMI) ≥ 30 kg/m^2^, can lead to secondary pathologies such as hypertension, metabolic syndrome, diabetes, insulin resistance, and inflammation, which increase the risk for cardiovascular (CV) and metabolic diseases (2–4).

CV risk varies with body fat distribution in both non-obese and obese individuals, highlighting the importance of examining body fat distribution alongside anthropometric parameters (5, 6). BMI alone does not distinguish fat mass from fat-free mass or characterize regional fat distribution. Composite indices combining anthropometric and biochemical information have therefore received increasing attention. For example, the visceral adiposity index combines BMI, waist circumference, triglycerides, and high-density lipoprotein cholesterol and has been proposed as a marker of visceral adipose dysfunction and cardiometabolic risk (7). Bioelectrical impedance analysis provides complementary, non-invasive estimates of specific body-composition compartments, including visceral fat (VF), total body fat (TBF), fat mass and fat-free mass. Although it is well established that the distribution of body fat has different effects on the CV system, there are only a few studies that have analyzed the association between body fat distribution and vascular damage such as arterial stiffness (5, 6, 8, 9). Their findings are inconsistent and the underlying physiological mechanisms are not well understood (5, 9, 10). Recent studies suggest that the interplay between unhealthy lifestyle factors and adverse biological conditions, such as elevated glucose levels, high blood pressure or obesity, may contribute to increased inflammation, which could elevate the risk of atherosclerosis. According to this hypothesis, proinflammatory markers could exacerbate arterial stiffness (8, 11, 12).

Inflammatory markers such as Tumor Necrosis Factor-alpha (TNF-α) and Interleukin-6 (IL-6) have been less frequently examined in this context (13–16). The Vascular cell adhesion molecule-1 (VCAM-1), primarily stimulated by TNF-α, serves as a marker of endothelial health, rather than general inflammation. While some studies suggest a potential relationship between VCAM-1 and arterial stiffness, research on this topic remains limited (16, 17). Findings on inflammation and arterial stiffness remain inconsistent due to factors like pre-existing diseases, age, sex, or racial differences, yet inflammatory markers are known to affect vascular health (18–22).

Arterial stiffness is an independent predictor of CV morbidity and mortality and can be assessed via pulse wave analysis, including pulse wave velocity (PWV), aortic augmentation index (AIx), and central pulse pressure (cPP) (23–25). Although AIx and cPP are meaningful indices, only a few studies have examined more indices than PWV. AIx is far more complex than PWV, and both can demonstrate divergent trends with ageing and inflammation. Thus, they should be viewed as complementary rather than interchangeable measures of arterial stiffness (26, 27).

Inflammation is itself an obesity-related cardiometabolic factor, however the extent to which selected inflammatory pathways explain associations between adiposity and arterial stiffness remains uncertain. Most previous studies of adiposity and arterial stiffness have relied on anthropometric measures, selected populations with severe obesity or cardiometabolic disease, and PWV as the principal vascular outcome (6, 8, 9, 12, 15, 28). Consequently, it remains uncertain whether BIA-derived fat measures are associated with complementary indices of aortic stiffness and wave reflection in a relatively young and generally healthy population, and whether selected inflammatory markers explain a part of these associations. Clarifying these cross-sectional patterns can prioritize mechanisms and measurements for longitudinal research, although it cannot establish causality or clinical utility.

The objective of this cross-sectional study was to quantify the associations of bioimpedance-derived VF, TBF, fat mass index (FMI), and fat-free mass index (FFMI) with carotid-femoral pulse wave velocity (cfPWV), AIx, and cPP, and to assess whether TNF-α, IL-6, or VCAM-1 act as intermediate factors in the observed associations.

## 2 Materials and methods

### 2.1 Study design and study population

The data used in this cross-sectional study were derived from the MEGA study, a population-based cohort study conducted between 2018 and 2021 by the Chair of Epidemiology, Medical Faculty, University of Augsburg, Germany. The main objective of the study was to investigate immunological particularities that are associated with body composition, anthropometric measures, diet and general lifestyle (e.g., physical activity, etc.). Therefore, the best possible population-based sample of participants was recruited including normal-weight individuals as well as individuals with higher BMI. The recruitment of participants was done in various ways, which were posters and flyers (e.g., at doctor's offices), announcements in newspapers and on social media, direct recruitment of participants in public places (e.g., shopping mall, university hospital), and in later stages by word of mouth. A total of 238 healthy, non-obese, and obese adult participants, aged between 24 and 70 years (31.1% men and 68.9% women), were examined at least three times over a period of nine months. The present study utilized the baseline measurements.

Participants with one of the following conditions were excluded: Use of antibiotics or corticosteroids up to three months prior to the baseline visit, current use of immunosuppressive medicine, history of significant illness and acute febrile illness.

All study participants gave written informed consent. The study was approved by the Ethics Committee of the Ludwig-Maximilians-Universität München (reference number 18–637) and was performed in accordance with the Declaration of Helsinki. The study was registered at the German Register of Clinical Studies (DRKS) DRKS00015784.

### 2.2 Data collection

In addition to various physical examinations and face-to-face interviews performed by physicians and trained study nurses, participants were required to complete several questionnaires regarding medication and dietary intake, health and well-being, sleep quality, eating disorders, smoking behavior, etc.

Habitual physical activity was assessed using the short version of the International Physical Activity Questionnaire (IPAQs) (29). To assess the alcohol consumption frequency, the short version of the Alcohol Use Disorders Identification Test (AUDIT-C) was used (30).

All participants were instructed to maintain an overnight fasting period of at least 10 h. Participants were allowed to take their medication as usual on the morning of the examination with a sip of water. Consumption of coffee and physical activity on the morning of the examination were not permitted. All examination procedures were conducted in accordance with predefined standard operating procedures.

### 2.3 Anthropometric and BIA measurements

Anthropometric measurements, including height, weight and waist circumference (WC), were taken with participants wearing light clothing and no shoes. WC was measured in the middle between the lower rib and the iliac crest. BMI was calculated as weight (in kilograms) divided by height (in square meters). To estimate body composition, a multifrequency bioelectrical impedance analysis (BIA) was conducted using the SECA mBCA515 device. For the measurement, participants stood barefoot on the platform of the device and held the handrail at a position appropriate for their height. The device uses an 8-point electrode configuration, comprising four pairs of electrodes: two pairs integrated into the handrail grips and two pairs integrated into the platform.

It is a non-invasive method that measures raw impedance parameters, including resistance, reactance, and phase angle across different body segments. Based on these measurements and body weight, the device estimates body composition parameters, such as VF (in liters), TBF (in % of total body weight), FMI (in kg/m^2^), and FFMI (in kg/m^2^). The calculations are performed using proprietary, clinically validated algorithms developed by SECA, which incorporate bioelectrical impedance measurements together with anthropometric and demographic variables, including body weight, height, age, sex, and ethnicity.

### 2.4 Measurement of arterial stiffness and blood pressure

To measure and characterize arterial stiffness, pulse wave was assessed non-invasively using the Vicorder system (SMT medical technology GmbH & Co. KG, Würzburg, Germany), which derives the arterial pressure waveform using an oscillometric technique. Blood pressure cuffs are placed around the neck and upper thigh to record the pulse waves and determine the speed (i.e., ratio of the measured transit time and the measured distance) of the pulse wave between the Arteria carotis communis and the Arteria femoralis. Inelastic arteries transmit the pulse wave faster than healthy, highly elastic arteries.

There was a rest period of ten minutes in a lying position before this examination. Additionally, blood pressure was measured at the end of the interview, after the participants had been sitting quietly for at least five minutes. Three blood pressure measurements with a rest interval of two minutes in between were taken on the right arm.

cfPWV is considered the gold standard for determining arterial stiffness (31–33).

The AIx represents the contribution of wave reflections to the rise in central systolic pressure and pulse pressure. It measures the extent to which reflected waves from peripheral arteries augment the central aortic pressure during systole. In addition to arterial stiffness, this parameter also depends on sex, body size, heart rate, left ventricular function, and peripheral resistance (32).

Central pulse pressure (cPP) refers to the difference between peak pressure in the aorta during systole and the lowest pressure during diastole. cPP provides insights into the pressure exerted on the large arteries close to the heart and is therefore considered a more accurate indicator of CV risk than peripheral pulse pressure (32).

Published validation data indicate good repeatability of PWV measurements obtained using oscillometric techniques (34). Vicorder and magnetic resonance imaging were shown to perform similarly in detecting age-related changes in pulse wave velocity (35). In a study assessing the reproducibility of the Vicorder, repeated measurements of AIx showed a correlation coefficient of *r* = 0.736, with a mean difference between measurements of 0.72 ± 4.86 percentage points. Repeated measurements of PWV showed a correlation coefficient of *r* = 0.96 and a mean difference of 0.03 ± 0.36 m/s (36).

### 2.5 Biomarker analyses

Blood samples were collected in an overnight fasting state of at least 10 h. At the University Hospital of Augsburg, standard laboratory parameters, including blood lipids were measured immediately from serum. EDTA plasma samples were analyzed using the Olink inflammation panel. Information on this panel can be found at the website of Olink Proteomics (https://olink.com/products/olink-target-96). The procedure of measurements is based on the Proximity Extension Assay (PEA) which was described in a prior publication (37).

Before examining associations with arterial-stiffness outcomes, TNF-α, IL-6, and VCAM-1 were selected based on their literature-supported relevance to adiposity, vascular inflammation, endothelial activation, and arterial stiffness, as well as the availability of valid measurements for the planned analyses. Selection was not based on observed outcome associations, effect estimates, or *p*-values. Valid measurements were available for 200 participants for TNF-α, 103 for IL-6, and 206 for VCAM-1.

### 2.6 Statistical analyses

Participants with missing BIA or pulse wave analysis measurements (*n* = 19) were considered to be completely at random and thus excluded, resulting in a final sample of *n* = 219 participants. Baseline characteristics were calculated for the total sample and separately for BMI < 30 kg/m^2^ and BMI ≥ 30 kg/m^2^. Depending on the distribution, descriptive data are expressed as mean and standard deviation or as median and 25th-75th percentile for continuous variables, and as the number of participants and percentage for categorical variables.

The bivariate relationships between the BIA measurements, anthropometric characteristics and the arterial stiffness indices were examined using Pearson's correlation coefficient, while Spearman's correlation was applied for visceral fat due to the presence of outliers.

To examine the association between BIA measurements (independent variables) and arterial stiffness indices (dependent variables), multivariable linear regression models were conducted. All models were adjusted for age, sex, hypertension, heart rate, diabetes, smoking, physical activity, risky alcohol consumption, LDL-cholesterol, and intake of antihypertensive drugs, based on existing literature (19, 38). Additionally, as part of a sensitivity analysis, anthropometric measurements, including BMI and WC, were also examined. Given the number of independent variables included in the models, the adjusted R^2^ values were reported as a measure of the explained variance. The required assumptions (normal distribution, no influential outliers, and independence as well as homoscedasticity of residuals) were evaluated in all models. The linearity assumption was evaluated by adding second-order polynomials and the absence of multicollinearity was confirmed by the calculation of the variance inflation factor.

Due to heteroskedasticity of the residuals, all *p*-values and confidence intervals (CIs) were calculated based on robust standard errors, using the HC3 adjustment. To lower the false discovery rate (FDR) all *p*-values were adjusted for multiple comparisons using the Benjamini-Hochberg procedure.

We reported the regression coefficients with 95% CIs. The significance level for all statistical tests was set at *α* = 0.05. All statistical analyses were performed with IBM SPSS Statistics Software version 27.0 (IBM Corp., Armonk, NY) and figures were generated in R version 4.6.1.

### 2.7 Mediation analysis

To examine whether the selected inflammatory markers explain part of the observed associations between the BIA and anthropometric measurements and arterial stiffness indices, mediation analyses were performed using PROCESS v4.3. Given the cross-sectional study design, these analyses were used to quantify indirect associations and in the following the inflammatory markers are referred to as potential intermediate factors.

TNF-α, IL-6, and VCAM-1 had been selected before conducting analyses involving the arterial stiffness outcomes, based on their biological and literature-supported relevance and the availability of valid measurements in a sufficient number of participants for the planned analyses. The selection of the markers was not based on observed associations with arterial stiffness, effect estimates, or *p*-values. Each marker was evaluated separately as a potential explanatory variable. Only exposure-outcome pairs that showed significant associations in the multivariable linear regression analyses prior to applying the FDR correction were carried forward to the statistical mediation analyses. Bootstrapping with 5,000 samples together with the heteroscedasticity-consistent inference method HC3 were employed to compute the 95% CIs and inferential statistics.

In the statistical mediation analyses, the direct, indirect, and total effects were estimated (Figure 1). The direct effect (c') represents the exposure-outcome association conditional on the selected inflammatory marker. The indirect effect (a_*_b) is calculated as the product of the exposure-marker association (a) and the marker-outcome association conditional on the exposure (b). The total effect (c) represents the overall association between the exposure and the arterial stiffness outcome. In this cross-sectional study, these model-based estimates were interpreted as associations and not as evidence of causal pathways.

**Figure 1:**
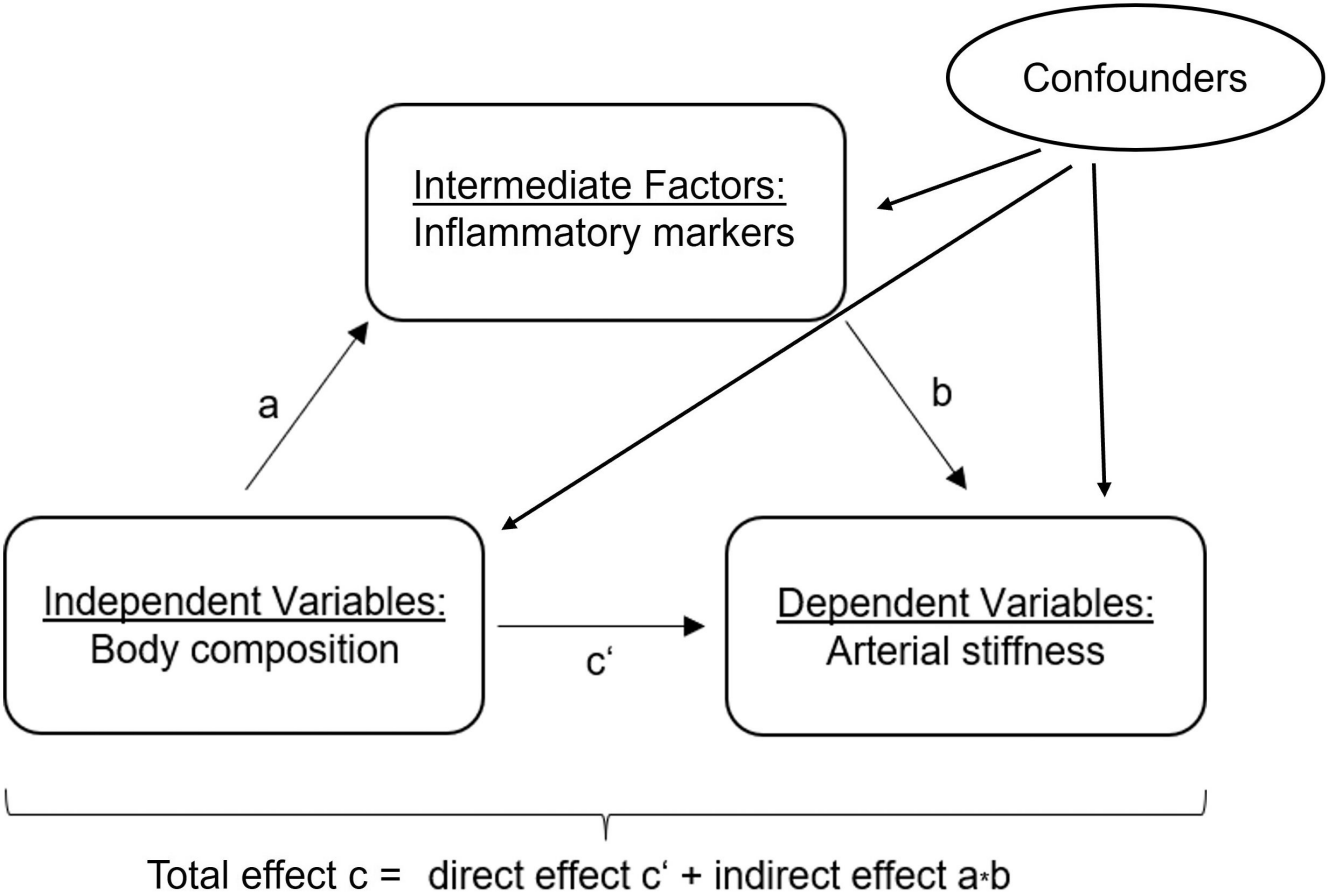
Regression framework for the investigation of the association between body composition (visceral fat, total body fat, fat mass index, body mass index, waist circumference) and arterial stiffness (augmentation index, central pulse pressure) including inflammatory markers (tumor necrosis factor-alpha, human vascular cell adhesion molecule 1, interleukin-6) as potential intermediate factors. Measured confounders included in the models: age, sex, hypertension, heart rate, diabetes, smoking, physical activity, alcohol use disorder, LDL-cholesterol, intake antihypertensives. Arrows represent model-based regression paths. Unmeasured and residual confounding, bidirectional associations as well as reverse causation are not displayed but cannot be ruled out in this cross-sectional study design.

## 3 Results

### 3.1 Baseline characteristics

Baseline characteristics stratified by BMI (obese and non-obese) are presented in Table 1. The sample consisted of 219 participants (148 women) with a median age of 48 years (25th-75th percentile: 35-56 years). Of these, 137 participants (102 women) had a BMI < 30 kg/m^2^, while 82 participants (46 women) had a BMI ≥ 30 kg/m^2^. Pre-existing hypertension was present in 26.5% of the participants and 14.2% reported intake of anti-hypertensive medication.

**Table 1:** Baseline characteristics of participants by BMI < 30 kg/m^2^ and BMI ≥ 30 kg/m^2^.

Variable	Total (*n* = 219)	BMI < 30 kg/m^2^ (*n* = 137)	BMI ≥ 30 kg/m^2^ (*n* = 82)
Age	48 (35-56)	48 (35-56)	46 (36-57)
Female sex (%)	148 (67.6)	102 (74,5)	46 (56.1)
Body mass index [kg/m^2^]	25.2 (22.7-32.8)	23.2 (21.2-24.9)	34.8 (32.2-39.9)
Body height [cm]	170.9 (9.2)	170.3 (8.6)	171.9 (10.2)
Body weight [kg]	76.0 (64.3-100.3)	66.2 (60.0-74.7)	105.2 (97.4-117.9)
Waist circumference [cm]	87.0 (77.0-106.0)	79.0 (73.5-86.0)	112.5 (104.0-121.5)
Systolic blood pressure [mmHg]	118.6 (16.1)	112.8 (13.6)	128.5 (15.2)
Diastolic blood pressure [mmHg]	78.6 (10.8)	74.6 (9.2)	85.4 (9.8)
cfPWV Pulse wave velocity [m/s]	7.6 (1.3)	7.5 (1.3)	7.9 (1.4)
AIx Augmentation index [%]	20.9 (8.2)	19.9 (8.0)	22.5 (8.2)
cPP Central pulse pressure [mmHg]	61.0 (53.0-72.5)	60.0 (52.5-71.0)	62.0 (53.3-74.5)
Heart rate (bpm)	60 (55-68)	59 (54-68)	63 (56-70)
Visceral fat [L]	1.4 (0.7-3.4)	0.9 (0.5-1.3)	4.2 (2.6-6.0)
Total body fat [%]	33.5 (10.3)	28.4 (8.0)	42.0 (7.8)
Fat mass index [kg/m2]	8.5 (5.9-13.2)	6.7 (5.3-8.3)	15.2 (12.0-18.0)
Fat-free mass index [kg/m2]	17.6 (15.6-20.4)	15.9 (15.0-17.7)	20.6 (18.8-22.9)
Triglycerides [mg/dL]	81.0 (59.0-123.5)	72.0 (56.0-97.0)	113.5 (78.8-150.3)
LDL Cholesterol [mg/dL]	120.0 (31.5)	118.1 (31.2)	123.1 (32.0)
HDL Cholesterol [mg/dL]	60.0 (48.0-72.5)	66.0 (54.0-81.0)	51.0 (43.8-60.3)
Total cholesterol [mg/dL]	186.4 (36.0)	188.0 (36.0)	183.9 (36.2)
Smoking			
Never (%)	107 (48.9)	80 (58.4)	27 (32.9)
Previous (%)	82 (37.4)	44 (32.1)	38 (46.3)
Current (%)	30 (13.7)	13 (9.5)	17 (20.7)
Risky alcohol consumption (AUDIT-C)			
Negative (%)	111 (50.7)	67 (48.9)	44 (53.7)
Positive (%)	108 (49.3)	70 (51.1)	38 (46.3)
Physical Activity (IPAQ)			
High (%)	125 (57.1)	87 (63.5)	38 (46.3)
Moderate (%)	59 (26.9)	37 (27.0)	22 (26.8)
Low (%)	35 (16.0)	13 (9.5)	22 (26.8)
Pre-existing diseases			
Diabetes (%)	9 (4.1)	4 (2.9)	5 (6.1)
Hypertension (%)	58 (26.5)	19 (13.9)	39 (47.6)
Heart Failure (%)	1 (0.5)	0 (0)	1 (1.2)
Cardiac Infarction (%)	2 (0.9)	1 (0.7)	1 (1.2)
Use of anti-hypertensives (%)	31 (14.2)	9 (6.6)	22 (26.8)
Inflammatory markers			
TNF-α [pg/mL]	53.7 (22.5-65.5)	50.6 (22.5-64.6)	57.3 (22.8-65.6)
IL-6 [pg/mL]	2.2 (1.9-2.5)	2.1 (1.9-2.4)	2.3 (1.9-2.5)
VCAM-1 [pg/mL]	1,003,000.0 (811,196.8–2,080,775.0)	990,335.8 (796,832.3–2,021,975.0)	1,044,250.0 (833,224.9–2,154,400.0)

Values are expressed as mean (standard deviation), median (25th-75th percentile) or number (%) BMI, Body mass index; LDL, Low-density lipoprotein; HDL, High-density lipoprotein.

### 3.2 Correlations between BIA measurements, arterial stiffness indices, and inflammatory markers

All BIA measurements, except for FFMI, were significantly correlated with cfPWV, AIx, and cPP (Figure 2, Supplementary Table S1). Among these, VF demonstrated the strongest correlation with cfPWV (*ρ* = 0.305, *p* < 0.001), whereas TBF exhibited the strongest correlation with AIx (*r* = 0.358, *p* < 0.001).

**Figure 2:**
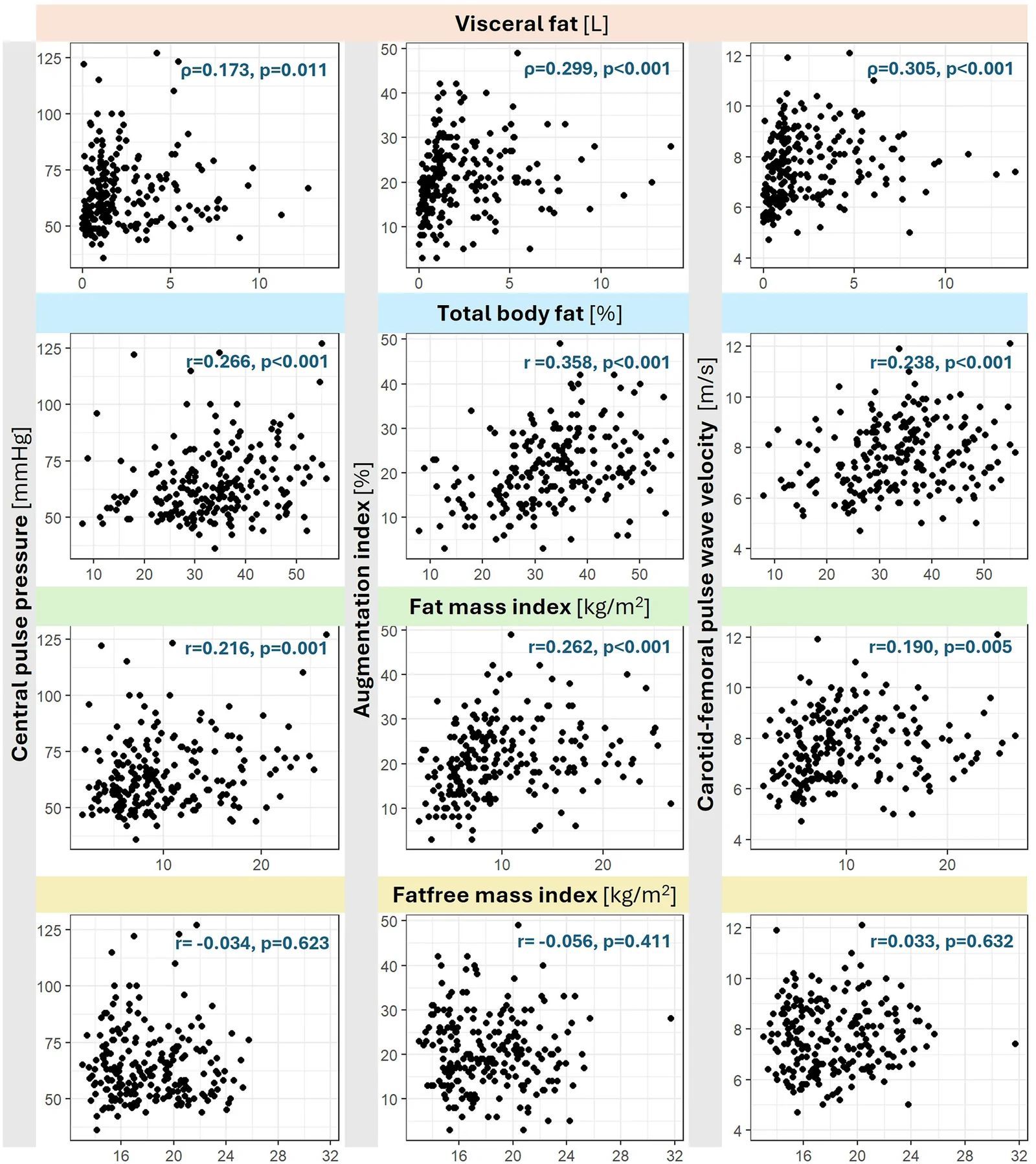
Scatterplots depicting the bivariate relationships between BIA measurements (visceral fat, total body fat, fat mass index, and fatfree mass index) and indicators for arterial stiffness (augmentation index, carotid-femoral pulse wave velocity, and central pulse pressure). Correlation coefficients are reported as Pearson's r or Spearman's *ρ*.

Both BMI and WC showed a weak but significant correlation with cfPWV (*r* = 0.152, *p* = 0.025 and *r* = 0.179, *p* = 0.008) and AIx (*r* = 0.168, *p* = 0.013 and *r* = 0.172, *p* = 0.011), whereas cPP was significant only for BMI (*r* = 0.146, *p* = 0.033). Correlations between BIA measurements and inflammatory markers were not statistically significant (Supplementary Figure S1).

### 3.3 Associations between BIA measurements and arterial stiffness indices

Figure 3 presents the associations between various BIA measurements and arterial stiffness indices derived from multivariable linear regression analyses. VF, TBF, and FMI were positively associated with AIx. The adjusted coefficients were *β* = 0.642 (95% CI: 0.172–1.112, *p* = 0.032) for VF, *β* = 0.186 (95% CI: 0.083–0.288, *p* < 0.001) for TBF, and *β* = 0.333 (95% CI: 0.157–0.508, *p* < 0.001) for FMI. A positive association between FMI and cPP (*β* = 0.550, 95% CI: 0.002–1.097, *p* = 0.049) was observed prior to adjusting the *p*-values for multiple testing; however, this association lost its statistical significance after the FDR correction was applied. The results on AIx remained significant upon sensitivity analysis with BMI (*p* = 0.001) and WC (*p* = 0.002) in the models (Supplementary Table S2).

**Figure 3:**
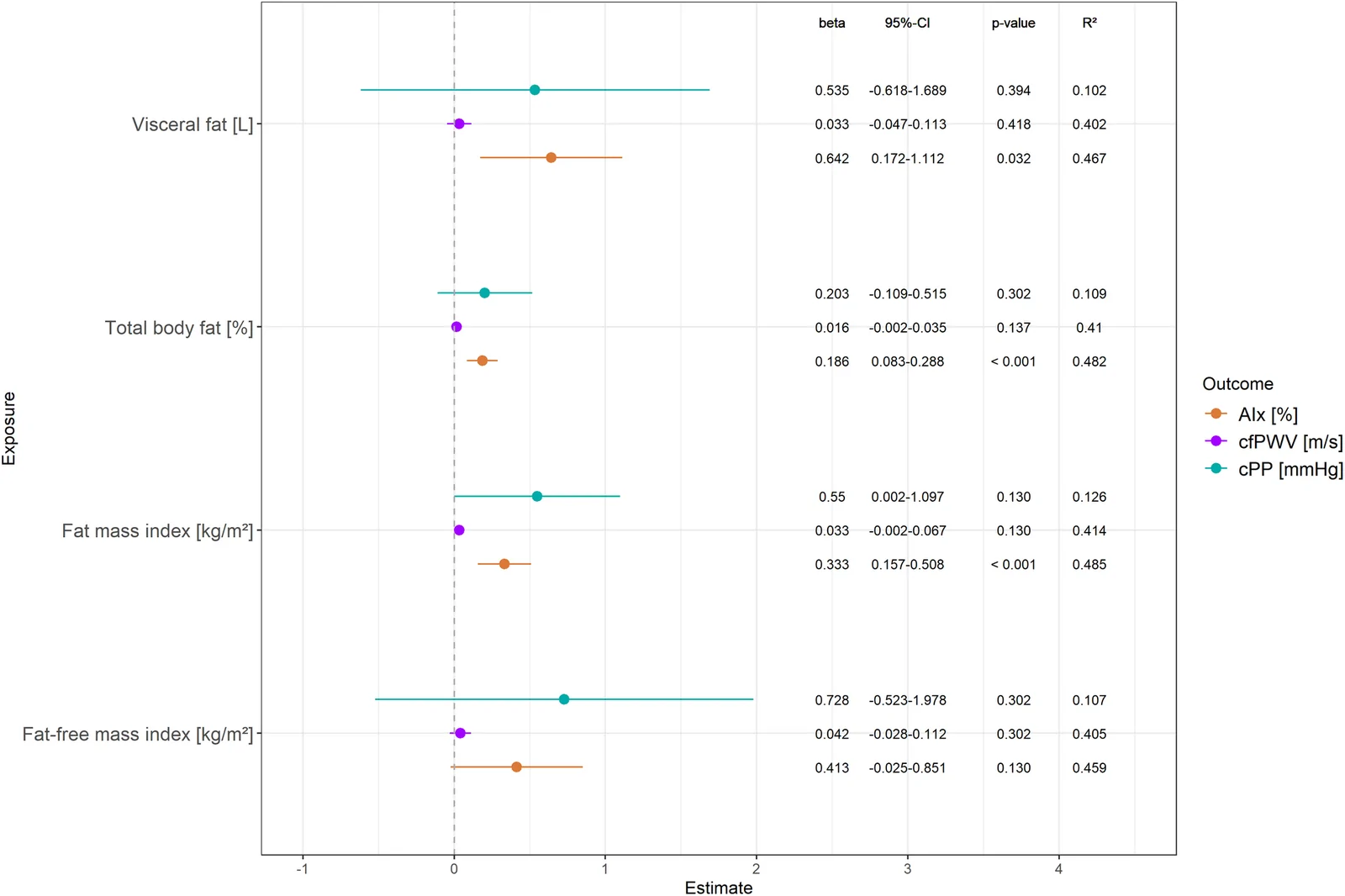
Multivariable linear regression analysis for the association between BIA measurements and arterial stiffness indices (cfPWV: *n* = 210; AIx: *n* = 212; cPP: *n* = 206). All models were adjusted for age, sex, hypertension, heart rate, diabetes, smoking, physical activity, alcohol use disorder, LDL-cholesterol, intake antihypertensives. All *p*-values and CIs are based on calculated robust standard errors. FDR-adjusted *p*-values and adjusted R^2^ values are reported. BIA, Bioelectrical impedance analysis; AIx, Augmentation index; cfPWV, Carotid-femoral pulse wave velocity; cPP, Central pulse pressure; CI, Confidence interval.

### 3.4 Indirect associations involving selected biomarkers

TNF-α, IL-6, and VCAM-1 were evaluated individually as potential intermediate factors for the exposure-outcome associations carried forward from the multivariable linear regression analyses involving AIx or cPP. In all models, the 95% bootstrap confidence intervals for the estimated indirect associations included zero, providing no statistical evidence that the selected inflammatory markers explained a notable proportion of the observed associations (Supplementary Table S3).

## 4 Discussion

In this cross-sectional analysis of 219 participants, higher VF, TBF, and FMI were associated with higher AIx, after multivariable adjustment and false discovery rate correction, whereas none of the BIA-derived measures remained associated with cfPWV or cPP. We found no evidence that the three selected inflammatory biomarkers explained part of the observed associations. This finding suggests, that adiposity may be more closely related to wave reflection, than to structural aortic stiffness in this cohort.

### 4.1 Body fat and pulse wave velocity

In the multivariable regression models, no significant associations between body composition parameters and cfPWV were observed. This may be attributed to differences in study populations and outcome measures, as our cohort included relatively healthy, non-elderly individuals which were predominantly non-obese (62.6%) and female (67.6%). Vascular changes due to adiposity may become more pronounced with advancing age, which could explain the lack of significant associations.

Most studies have examined the relationship between body composition, mostly assessed by anthropometric measures, and arterial stiffness in morbidly obese participants (6), or those with preexisting diseases (23). For instance, WC has been associated with PWV progression of the trunk in adolescents with Type 1 diabetes (20) and VF with carotid artery stiffness in patients with well-controlled, uncomplicated Type 2 diabetes (39). However, Tang et al., investigating a healthy cohort, similarly found that BMI was positively correlated with PWV but not an independent predictor of arterial stiffness. These findings align with our results, as we also studied a healthy cohort with a comparable mean age.

Several studies have highlighted sex-specific differences in the relationship between body fat and arterial stiffness (40). TBF was linked to arterial distensibility only in men (41), while Gong et al. identified it as a predictor of arterial stiffening in women over 60 years of age (10). PWV increases with age and is significantly higher among men (40), suggesting hormonal and vascular differences, including estrogen-mediated vascular relaxation and arterial tree length (42).

Some studies found paradoxical inverse associations between obesity markers and PWV (9, 15, 42), possibly due to differences in age, sex, obesity levels, health status, and the varying inclusion of confounding factors, such as the use of antihypertensive medications, blood pressure levels, or the presence of diabetes (9).

Additionally, many studies focused on brachial-ankle PWV (9, 40, 42) or carotid intima media thickness (19, 22, 43), limiting direct comparisons, as few have examined AIx or cPP as outcomes (44–46).

### 4.2 Body fat and augmentation index

After adjusting for confounders, the associations between BIA measurements and cfPWV were no longer statistically significant, while those with AIx remained robust after FDR correction. This may reflect the distinct mechanisms of cfPWV and AIx. While cfPWV primarily measures structural stiffness influenced by age and blood pressure, AIx is influenced not only by arterial stiffness but also by functional changes in vascular tone, peripheral vascular resistance, heart rate, and the timing and magnitude of reflected pressure waves (26, 32). The outcome-specific findings may therefore reflect earlier functional vascular differences, although this interpretation requires confirmation in longitudinal studies.

Few studies on arterial stiffness in obesity have included assessments of arterial stiffness using measures of peripheral wave reflection, such as AIx, and to the best of our knowledge, no studies have investigated this relationship beyond anthropometric measurements by incorporating BIA measurements.

Research on AIx shows inconsistencies regarding sex differences and inverse associations. Some studies have reported that a lower BMI is associated with higher AIx in women (28), while others have found inverse associations between BMI, WC and AIx (47).

Consistent with our findings in sensitivity analysis with BMI, a previous study with stroke patients observed that AIx increases with higher BMI (48).

The existing literature indicates that the predictive value of AIx is particularly relevant for younger individuals, with AIx reaching a plateau after the age of 60 years (49). It may be a better predictor of arterial stiffness than cfPWV in individuals under 50 years of age (50), aligning with our cohort with a mean age of 46.2 ± 12 years. Janner et al. identified AIx as a key marker of CV disease in younger individuals, influenced by age, heart rate, height, and systolic blood pressure (51).

Our results indicate that VF, TBF and FMI were positively associated with AIx, whereas FFMI was not. Particularly TBF and FMI demonstrated a highly significant association with AIx. Although BMI and WC also showed significant associations with AIx in sensitivity analyses, BIA measurements provide compartment-specific estimates of body composition and may therefore complement BMI and WC when characterizing associations between adiposity and vascular function. Given the relatively young mean age of the cohort, these findings may be relevant to the investigation of early vascular changes in younger adults, although age-specific associations were not examined.

### 4.3 Arterial stiffness and inflammation

Contrary to our initial hypothesis, TNF-α, IL-6, and VCAM-1 did not reveal as intermediate factors regarding the observed associations between body composition and AIx, as all bootstrap confidence intervals for the estimated indirect associations included zero. This contrasts with Diamant et al. who found that IL-6, which originates partly from adipose tissue, and CRP mediated the association between visceral obesity and carotid stiffness (39). Conversely, Christen et al. reported no mediation of inflammatory markers CRP and glycoprotein acetyls in the associations between body fat and subclinical atherosclerosis in a healthy cohort with a mean age of 56 years (19).

Only few prior studies have thoroughly investigated whether markers of inflammation or endothelial activation explain a substantial proportion of the association between body composition and arterial stiffness. Nevertheless, biological plausibility exists because visceral adipose tissue is infiltrated by pro-inflammatory macrophages, and contributes to low-grade systemic inflammation through the secretion of cytokines such as IL-6 and TNF-α (10, 15, 52). Previous studies have found that IL-6 and TNF-α are positively correlated with AIx (27), with IL-6 identified as an independent predictor of increased AIx (53). Additionally, IL-6 and CRP have been recognized as significant contributors to arterial stiffness in younger and metabolically at-risk populations (13, 26).

The present study also evaluated whether VCAM-1, a marker of endothelial activation (54), acts as an intermediate factor for the observed associations between body composition and arterial stiffness. VCAM-1 is regarded as both a marker and a mediator of CV disorders and was found to be positively associated with BMI and WC (37) and arterial stiffness in patients with peripheral arterial disease, resistant hypertension, and individuals over 60 years of age (17).

The absence of statistically supported indirect associations may reflect differences in study populations, in cardiometabolic burden, age, biomarker selection and vascular outcomes. The relatively healthy nature of our cohort, the smaller sample available for IL-6 (*n* = 103) and the short circulating half-life of cytokines in the bloodstream may also have reduced the ability to detect indirect associations. These findings do not exclude the potential relevance of other processes not assessed in the present study, including oxidative stress or adipokine dysregulation (14, 16, 17, 26, 52).

### 4.4 Clinical implications

Early identification of CV risk in obese patients is crucial. The observed associations of VF, TBF, and FMI with AIx suggest that BIA-derived body-composition measures may provide additional information on body fat distribution beyond BMI and WC. However, these cross-sectional findings do not establish that integrating BIA into routine assessments could improve CV risk stratification or that reducing a particular fat compartment lowers arterial stiffness. Longitudinal studies are needed to determine whether detailed body-composition measures provide prognostic information and whether AIx can identify early vascular changes in younger adults.

### 4.5 Strengths and limitations

This study has several strengths. Arterial stiffness is a well-validated risk marker for CV morbidity and mortality, and cfPWV is considered the gold standard for assessing arterial stiffness (31). Moreover, we investigated not only cfPWV but also additional arterial stiffness indicators such as AIx and cPP, which have been examined in a limited number of studies (12, 15, 28).

In addition to examining complementary measures of arterial stiffness, the study evaluated whether three selected inflammatory markers explained part of the observed associations between body composition and arterial stiffness.

However, this study has several limitations. First, as this is a cross-sectional study, the causal relationship between obesity and arterial stiffness cannot be established and reverse causality cannot be excluded. Thus, the interpretation is hypothesis-generating rather than causal. Despite comprehensive adjustment, we cannot exclude residual or unmeasured confounding. Furthermore, residual variability related to individual hydration status cannot be completely excluded and may have introduced some measurement variability in the BIA-derived body-composition parameters.

The sample size was relatively small and included fewer male participants. Additionally, the study population was limited to individuals with a mean age of 46 years from the Augsburg region, and the findings may not be generalizable to other age or ethnic groups.

Generally, magnetic resonance imaging or dual- energy x-ray absorptiometry may provide more precise measurements of TBF compared to BIA (55).

Finally, the present findings are limited to three selected inflammatory markers, based on prior literature and data availability and therefore do not constitute a comprehensive evaluation of the complete inflammatory proteome. Moreover, the number of available measurements differed between markers, particularly for IL-6, which may have reduced statistical precision and the ability to detect small indirect associations.

## 5 Conclusion

The findings of this investigation revealed that higher visceral fat, total body fat, and fat mass index as measured by BIA were associated with higher AIx in a healthy population of southern Germany. However, no indirect association through TNF-α, IL-6, or VCAM-1 was found. These findings suggest an outcome-specific association with wave reflection. Future research should aim to explore broader biomarker panels, consider longitudinal designs and investigate additional explanatory pathways. Further validation in more diverse populations, including sex-specific analyses, is warranted.

## Data Availability

The raw data supporting the conclusions of this article will be made available by the authors, without undue reservation.
