# Supplementary material for: The association between body composition and arterial stiffness: investigating the influence of inflammatory markers
Source: Front Cardiovasc Med. 2026 Sep 21;13:1898248. doi: 10.3389/fcvm.2026.1898248 (PMC13634821; doi:10.3389/fcvm.2026.1898248)
Supplement: Supplementary file 1 [file Datasheet1.pdf]

Supplementary material

The association between body composition and arterial stiffness: Investigating the influence of inflammatory markers

Julia Neubauer[1,2], Dennis Freuer[2], Marlene Haupt[1,3], Jakob Linseisen[2], Christine Meisinger[2], Simone Fischer[2*]

[1] Faculty of Social Work, Health & Nursing, University of Applied Sciences Ravensburg-Weingarten, Leibnizstr. 10, 88250 Weingarten, Germany

[2] Epidemiology, Medical Faculty, University of Augsburg, Stenglinstraße 2, 86156 Augsburg, Germany

[3] Faculty of Applied Social Sciences, Munich University of Applied Sciences, Am Stadtpark 20, 81243 München, Germany

*Corresponding Author:
Dr. Simone Fischer
simone.fischer@med.uni-augsburg.de
Epidemiology, Medical Faculty, University of Augsburg, Stenglinstraße 2, 86156 Augsburg, Germany

Table S1 Pearson/Spearman correlations between BIA measures, anthropometric characteristics and arterial stiffness indices

	cfPWV [m/s]		AIx [%]		cPP [mmHg]	
	r	P-value	r	P-value	r	P-value
Visceral fat [L]	0.305	<0.001* [a]	0.299	<0.001* [a]	0.173	0.011* [a]
Total body fat [%]	0.238	< 0.001*	0.358	< 0.001*	0.226	< 0.001*
Fat mass index [kg/m²]	0.190	0.005*	0.262	< 0.001*	0.216	0.001*
Fat-free mass index [kg/m²]	0.033	0.632	-0.056	0.411	-0.034	0.623
Body Mass Index [kg/m²]	0.152	0.025*	0.168	0.013*	0.146	0.033*
Waist circumference [cm]	0.179	0.008*	0.172	0.011*	0.099	0.151

[a] Spearman Correlation

* Statistical significance with α = 0.05; Abbreviations: BIA=Bioelectrical impedance analysis; cfPWV=Carotid-femoral pulse wave velocity; AIx=Augmentation index; cPP=Central pulse pressure

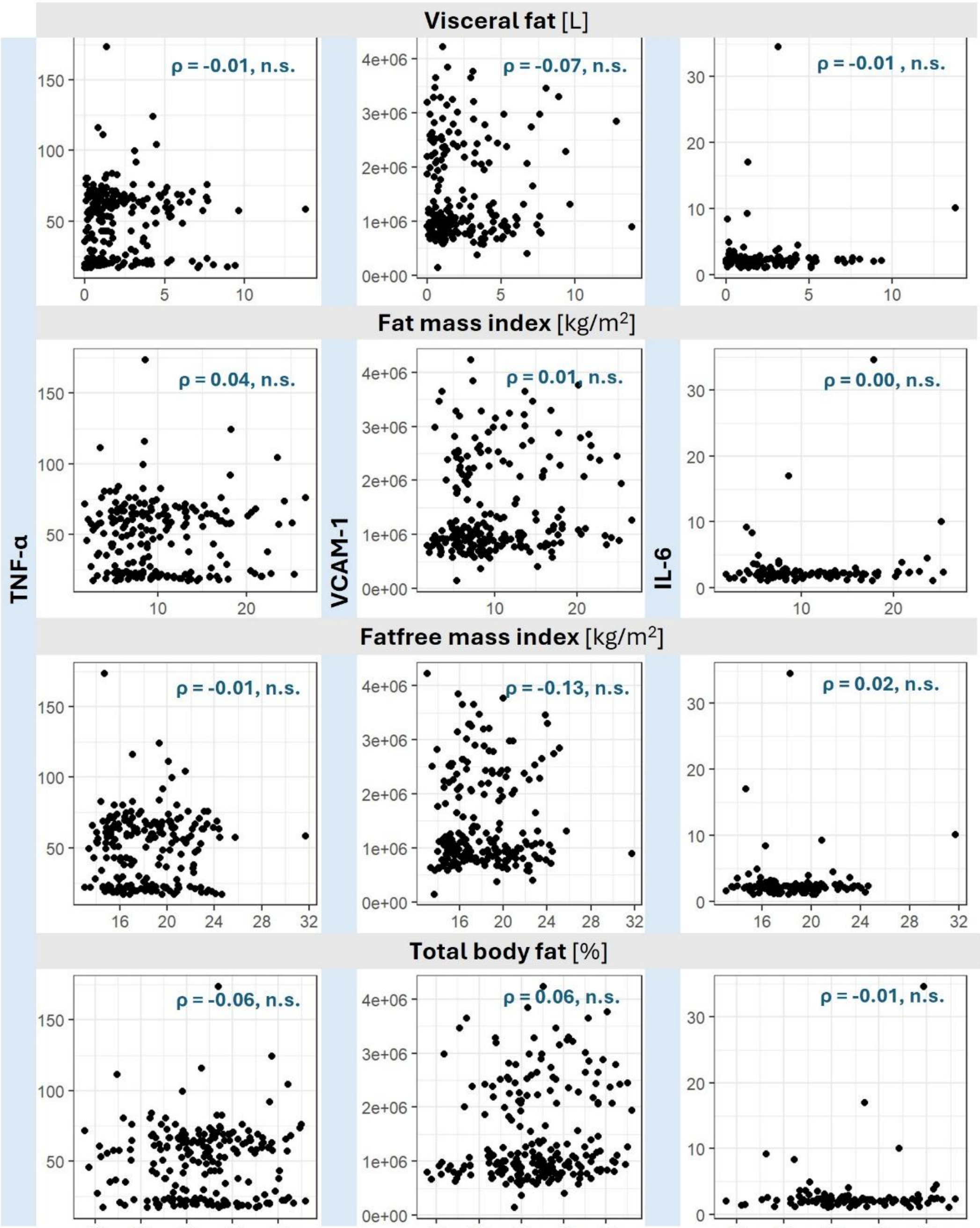

Figure S1 Scatterplots depicting the bivariate relationships between BIA measurements (Visceral fat, total body fat, fat mass index, and fatfree mass index) and inflammatory markers (TNF-α, VCAM-1, IL-6). Correlation coefficients are reported as Spearman's ρ. Abbreviation: n.s.=not significant.

Table S2 Multivariable linear regression analysis for the association between body mass index, waist circumference and arterial stiffness indices (cfPWV: n=210; AIx: n=212; cPP: n=206)

cfPWV [m/s]	β	95% CI	P-value	R^2
Body mass index [kg/m²]	0.022	-0.003-0.047	0.080	0.412
Waist circumference [cm]	0.008	-0.002-0.018	0.107	0.408
**AIx [%]**	**β**	**95% CI**	**P-value**	**R^2**
Body mass index [kg/m²]	0.223	0.090-0.357	0.001*	0.479
Waist circumference [cm]	0.093	0.034-0.152	0.002*	0.475
**cPP [mmHg]**	**β**	**95% CI**	**P-value**	**R^2**
Body mass index [kg/m²]	0.376	-0.026-0.778	0.066	0.122
Waist circumference [cm]	0.103	-0.041-0.247	0.161	0.107

All models were adjusted for age, sex, hypertension, heart rate, diabetes, smoking, physical activity, alcohol use disorder, LDL-cholesterol, intake antihypertensives; adjusted R^2 values were examined for each model; all p-values and CIs are based on calculated robust standard errors.
*Statistical significance with $\alpha = 0.05$
Abbreviations: cfPWV=Carotid-femoral pulse wave velocity; AIx=Augmentation index; cPP=Central pulse pressure; CI=Confidence interval

Table S3 Total, direct and indirect effects of BIA measurements, BMI and WC on AIx and cPP with inflammatory markers as potential mediators.

	TNF-α (n=200)		IL-6 (n=103)		VCAM-1 (n=206)	
	Coefficient	95% CI	Coefficient	95% CI	Coefficient	95% CI
AIx – Visceral fat						
Total effect	0.751**	0.186-1.316	0.718	-0.347-1.783	0.753**	0.254-1.252
Direct effect	0.765**	0.214-1.317	0.721	-0.356-1.799	0.793**	0.303-1.284
Indirect effect	-0.014	-0.101-0.055	-0.004	-0.249-0.046	-0.041	-0.137-0.022
AIx – Total body fat						
Total effect	0.202**	0.089-0.315	0.252**	0.096-0.407	0.196**	0.090-0.302
Direct effect	0.207**	0.095-0.319	0.251**	0.093-.0410	0.207**	0.101-0.312
Indirect effect	-0.005	-0.024-0.010	0.000	-0.034-0.011	-0.011	-0.034-0.004
AIx – Fat mass index						
Total effect	0.360**	0.159-0.562	0.371*	0.082-0.661	0.337**	0.156-0.158
Direct effect	0.366**	0.166-0.567	0.371*	0.074-0.668	0.353**	0.171-.0535
Indirect effect	-0.006	-0.041-0.020	0.000	-0.064-0.018	-0.016	-0.055-0.008
AIx – Body mass index						
Total effect	0.232**	0.079-0.385	0.195	-0.036-0.426	0.220**	0.082-.0358
Direct effect	0.236**	0.084-0.387	0.195	-0.042-0.432	0.229**	0.089-0.368
Indirect effect	-0.003	-0.025-0.016	0.000	-0.042-0.011	-0.009	-0.035-0.007
AIx – Waist circumference						
Total effect	0.097**	0.031-0.164	0.082	-0.012-0.175	0.096**	0.034-0.158
Direct effect	0.099**	0.035-0.163	0.081	-0.014-0.177	0.100**	0.040-0.161
Indirect effect	-0.002	-0.012-0.006	0.000	-0.016-0.005	-0.005	-0.017-0.003
cPP – Fat mass index						
Total effect	0.595	-0.058-1.248	0.315	-0.424-1.053	0.589	-0.017-1.194
Direct effect	0.615	-0.023-1.252	0.315	-0.429-1.059	0.639*	0.036-1.242
Indirect effect	-0.020	-0.107-0.060	0.000	-0.114-0.066	-0.050	-0.140-0.010

Values are beta coefficients (95% confidence intervals)
* $p \leq 0.05$, ** $p \leq 0.01$
Abbreviations: BIA=Bioelectrical impedance analysis; BMI=Body mass index; WC=Waist circumference; AIx=Augmentation index; cPP=Central pulse pressure, CI=Confidence interval
